# Supplementary material for: Succinate and its G-protein-coupled receptor stimulates osteoclastogenesis
Source: Nat Commun. 2017 May 31;8:15621. doi: 10.1038/ncomms15621 (PMC5460032; doi:10.1038/ncomms15621)
Supplement: Supplementary Information — Supplementary Figures and Supplementary Tables [file ncomms15621-s1.pdf]

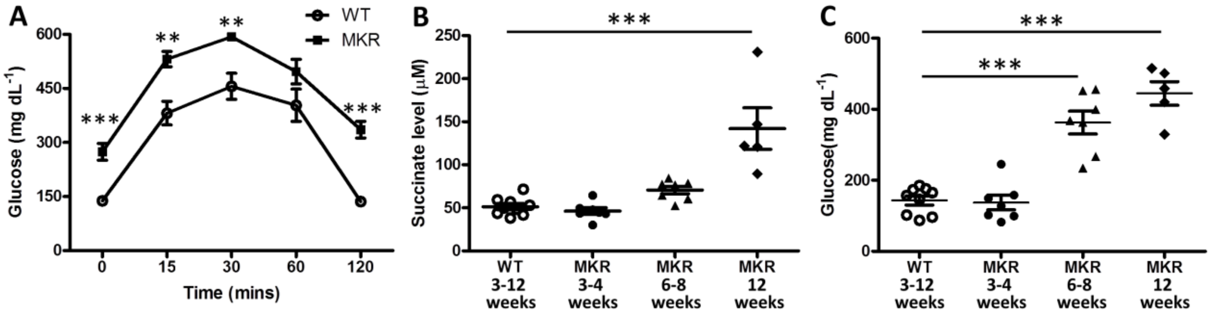

Supplementary Figure 1 (Related to Figure 1). Characterization of MKR mice. (**A**) Glucose tolerance test (GTT) after fasting. (**B**) Succinate levels and (**C**) glucose levels in WT (8~12 week-old) and MKR mice at 3 indicated age groups. *p<0.05, **p<0.005, ***p<0.001 by Bonferroni post hoc test after one-way ANOVA in comparison to WT.

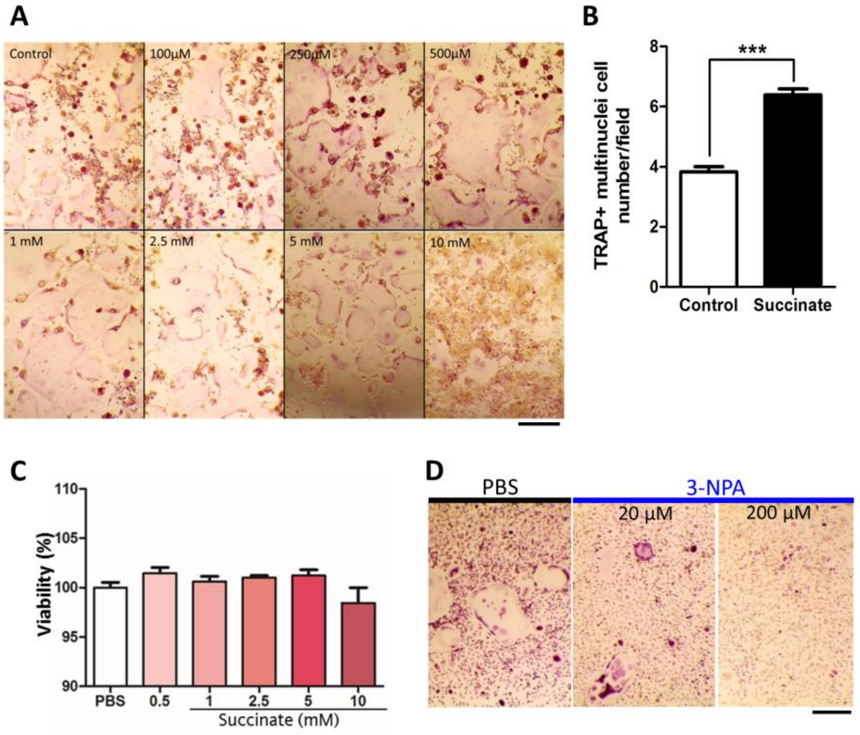

Supplementary Figure 2 (Related to Figure 2). Extracellular but not intracellular succinate stimulates osteoclastogenesis. Mouse bone marrow myeloid cells were cultured with Dulbecco's Modified Eagle Medium (DMEM) medium containing L-Glutamine and sodium pyruvate (Life technologies, Carlsbad, CA, USA), supplemented with 10% Fetal Bovine Serum (Atlanta Biologicals, GA, USA), 100 µg mL^{-1} streptomycin, 100 Units mL^{-1} penicillin (Gibco, Grand Island, NY, USA), 30ng ml^{-1} M-CSF and 50 ng ml^{-1} recombinant murine sRANK ligand (RANKL) (PeproTech, Rocky Hill, NJ, USA). Macrophage lineage cell RAW264.7 were purchased from ATCC (Catalog number TIB-71, Manassas, VA, USA). Cells were maintained in Dulbecco's Modified Eagle's Medium (DMEM) containing 10% FBS and incubated at 37°C in 5% CO2. The cells were seeded at 5,000 per well in 96-well-plate and stimulated with 50 ng ml-1 RANKL from the time of culture and fresh cytokines were added every other day. TRAP staining of PBS (Control) and succinate at indicated concentrations treated (**A**) RAW264.7 cells on day 5. Scale bar: 400 µm; (**B**) Data show mean±SEM of triplicated wells of succinate at 500µM. (**C**) Succinate treatment for 96-hours does not affect the viability of RAW264.7 cells. *** $p < 0.005$ by Bonferroni test post ANOVA, N=4. (**D**) 3-NPA suppresses osteoclastogenesis in osteoclasts. Scale bar: 250 µm.

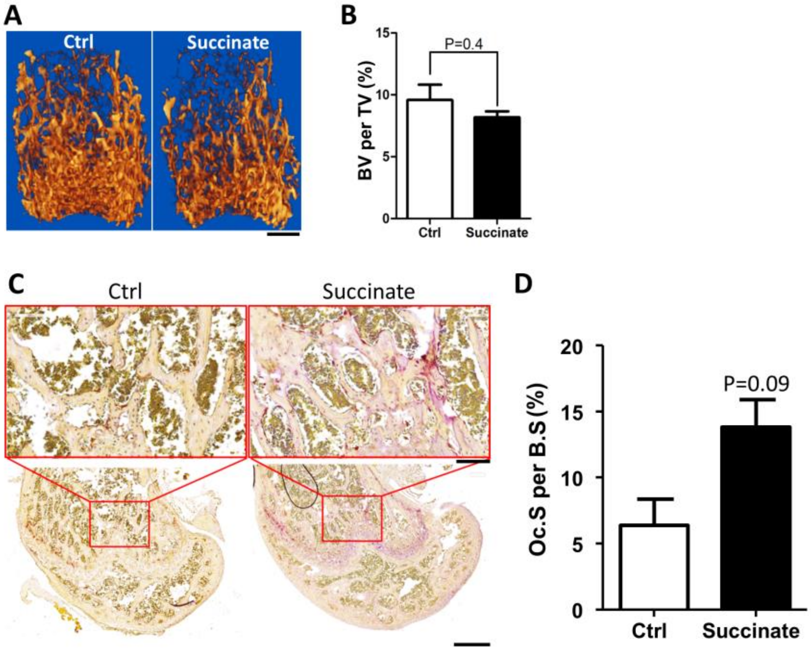

Supplementary Figure 3 (Related to Figure 3). Succinate administration stimulates osteoclastogenesis *in vivo*. (**A**) Representative µCT images (Scale bar: 500 µm) and (**B**) quantitation of femoral cancellous portion in wild type mice injected with control or succinate for 14-days. Data show Mean+SEM (N=3). (**C**) Representative TRAP staining (scale bars: Top 100 µm, bottom 500 µm) and (D) Numbers of TRAP+ cells in demonstrated area. Data were analyzed by two-tailed, unequal variance t-test.

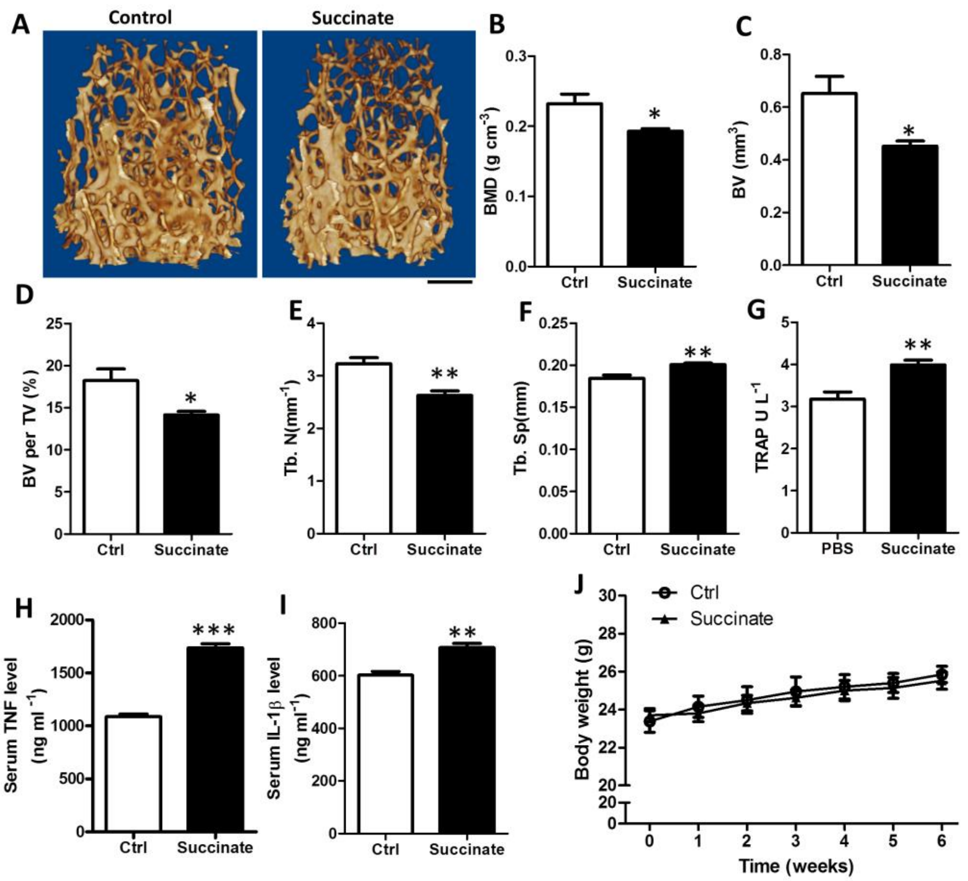

Supplementary Figure 4 (Related to Figure 3). Succinate administration reduced bone mass in C57/B6 strain WT mice *in vivo*. (**A**) Representative µCT images (Scale bar: 500 µm) and (**B**-**F**) quantitation of femoral cancellous portion in wild type mice injected with control or succinate for 6-weeks. Serum (**G**) TRAP5b, (**H**) TNF and (**I**) IL-1β levels. Data show Mean+SEM (N=5). Data were analyzed by two-tailed, unequal variance t-test, *p<0.05, **p<0.01.

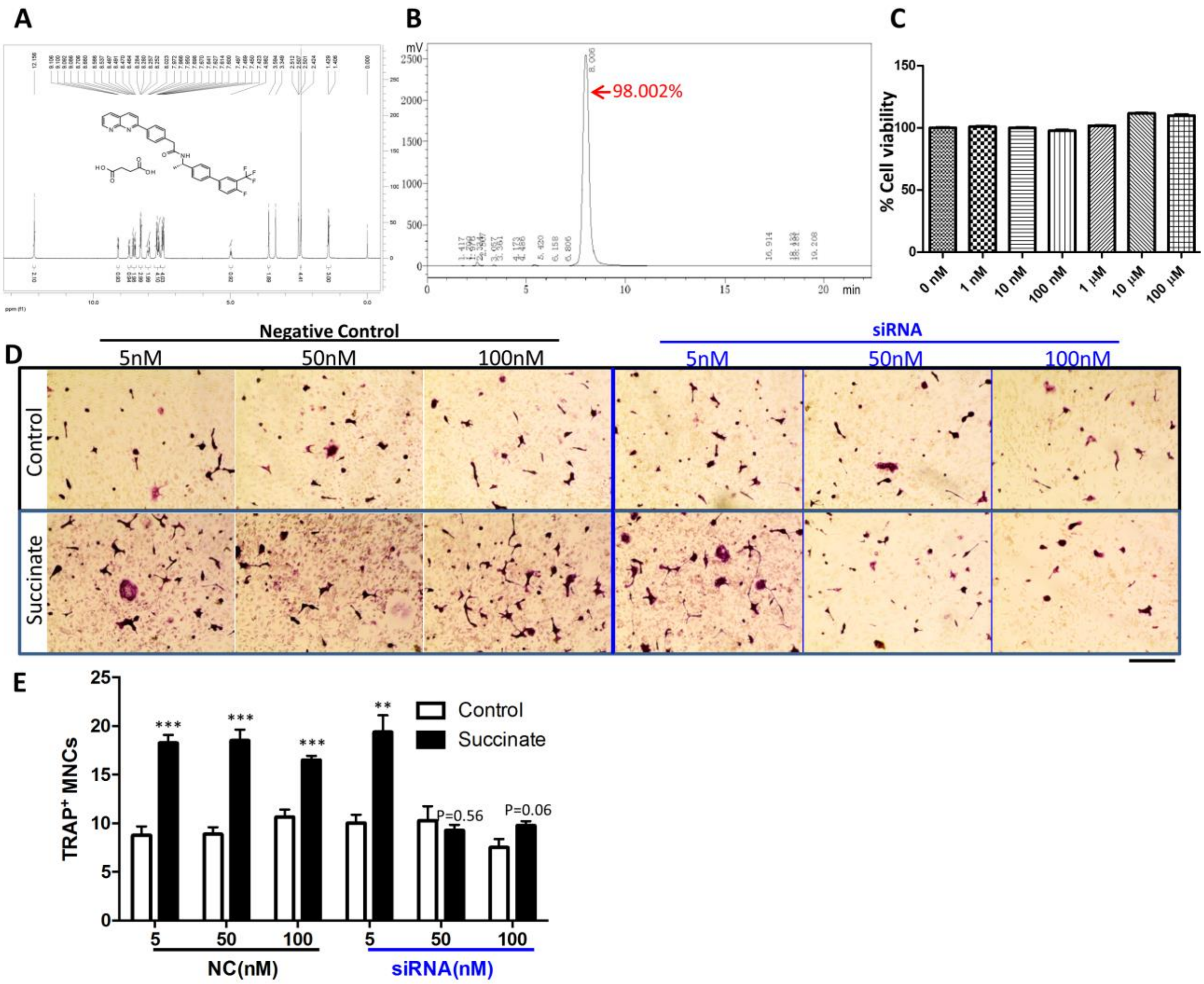

Supplementary Figure 5 (Related to Figure 4). SUCNR1 antagonist 4C. (**A**) Structure confirmed by Nuclear magnetic resonance spectroscopy (NMR) and (**B**) purity determined by HPLC of the synthesized compound. (**C**) No cytotoxicity of 4c to primary OC culture (without RANKL and MCSF). (**D**) Succinate stimulated osteoclastogenesis was blunted when SUCNR1 expression was suppressed by siRNA. Wild type of FVB strain mouse bone marrow cells were seeded in 96-well plate and stimulated with M-CSF (20 ng ml^{-1}) for 2 days to enrich osteoclast progenitors. SUCNR1 (GPR91) siRNAs or control siRNAs (Negative control, NC) were transfected using Lipofactamine-3000 (Invitrogen) on day 3 and again on day 6 to maintain the knockdown efficiency. Succinate or PBS was supplemented into the medium in 6 hours after each transfection and the osteoclast differentiation was assessed by TRAP staining. (**E**) Data show mean±SEM of triplicates. *** $p < 0.001$ according to Bonferroni post hoc test after ANOVA, N=4.

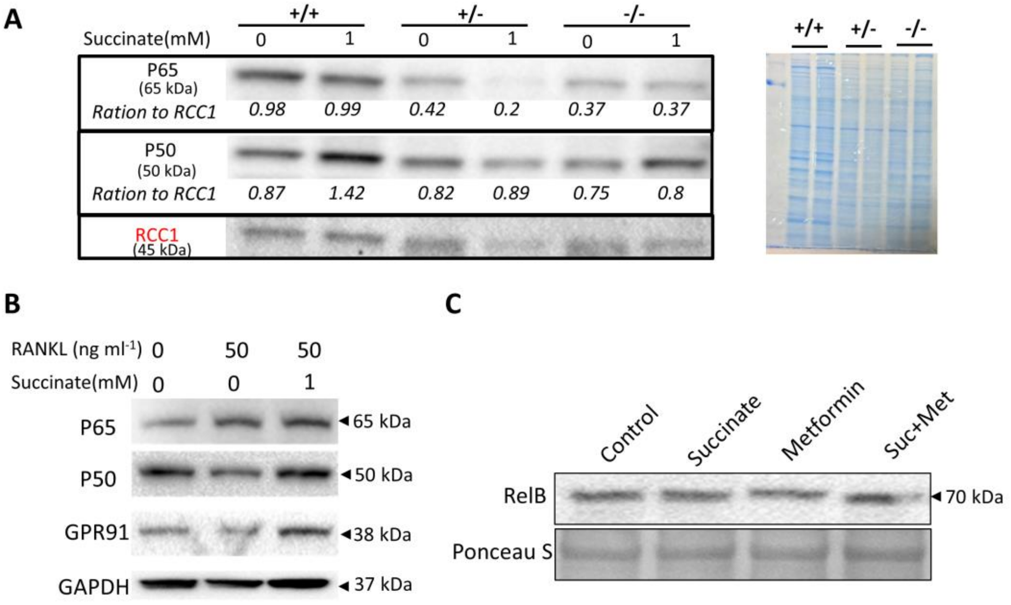

Supplementary Figure 6 (Related to Figure 6). Succinate induced osteoclastogenesis is dependent on canonical alterative NF- κB pathway. (**A**) Succinate increased nuclear p50 levels in osteoclasts derived from WT but not in SUCNR1 KO mice. Evaluation of nuclear P65, P50 and RCC1 levels in osteoclasts derived from WT (+/+), SUCNR1 heterozygous (+/-) and KO (-/-) mouse bone marrow cells by Western blot. Bone marrow cells were seeded in 10cm dish, non-attached cells were processed through Ficoll, seeded at 1.5×10^5 cells per ml and enriched by 20 ng ml^{-1} M-CSF for 48 hours and then induced to osteoclasts by 30ng ml^{-1} M-CSF and RANKL for 6 days. Cells were harvested for nuclear proteins with EpiGentek Nuclear extraction kit. (**B**) Succinate enhanced RANKL-induced NF- κB increase in RAW 264.7 cells. (**C**) Evaluation of RelB expression post succinate and metformin treatment in osteoclasts derived from MKR mouse bone marrow cells by Western blot, Ponseau S was used to show the protein loading amount.

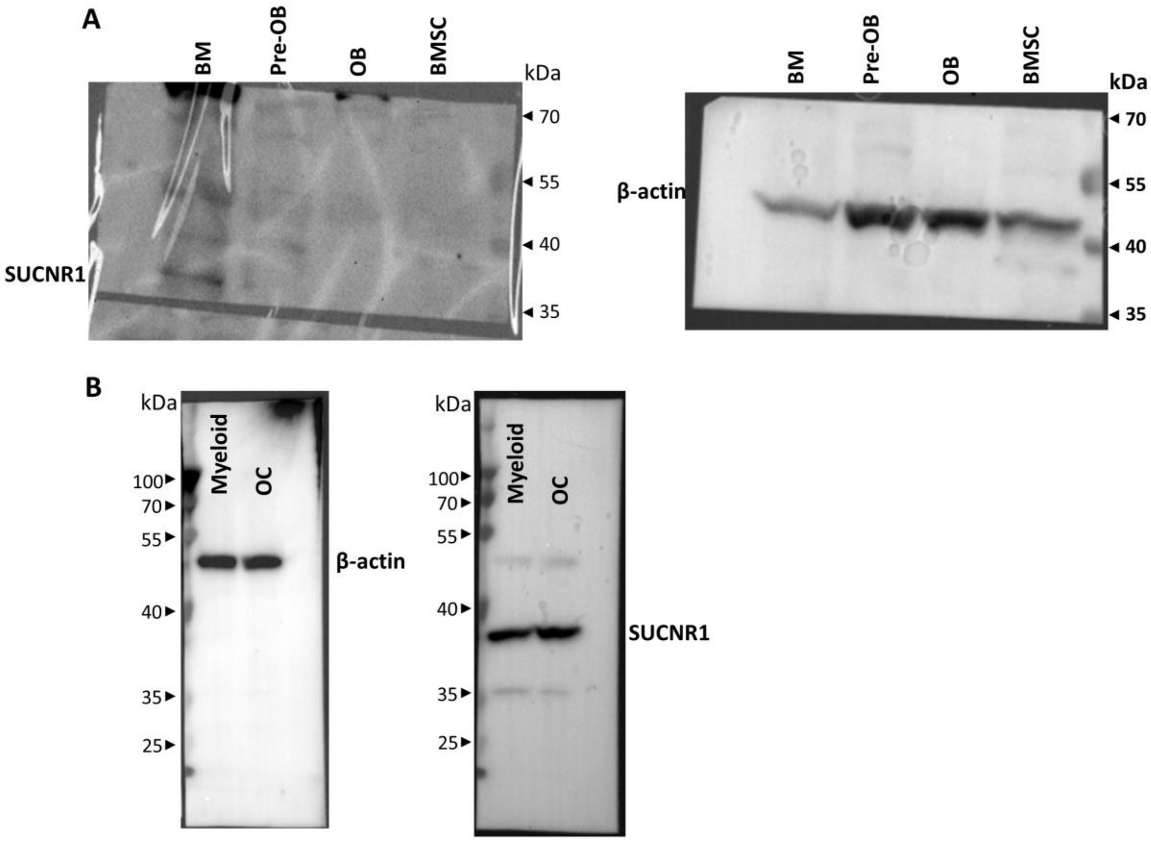

Supplementary Figure 7: Original western blot images used in Figure 4A**.** **(A)** Succinate-specific receptor SUCNR1 expression in bone marrow, stromal (BMSC) and osteoblast lineage cells (Pre-OB and OB). (**B**) Succinate-specific receptor SUCNR1 expression in hematopoietic lineage cells.

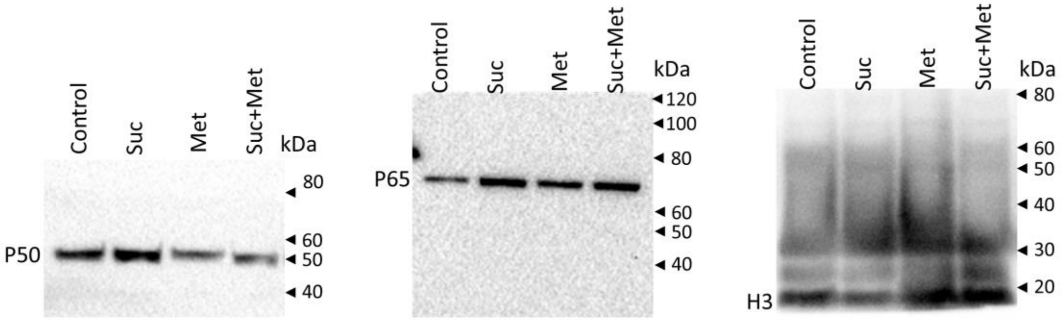

Supplementary Figure 8: Original western blot images used in Figure 6C.

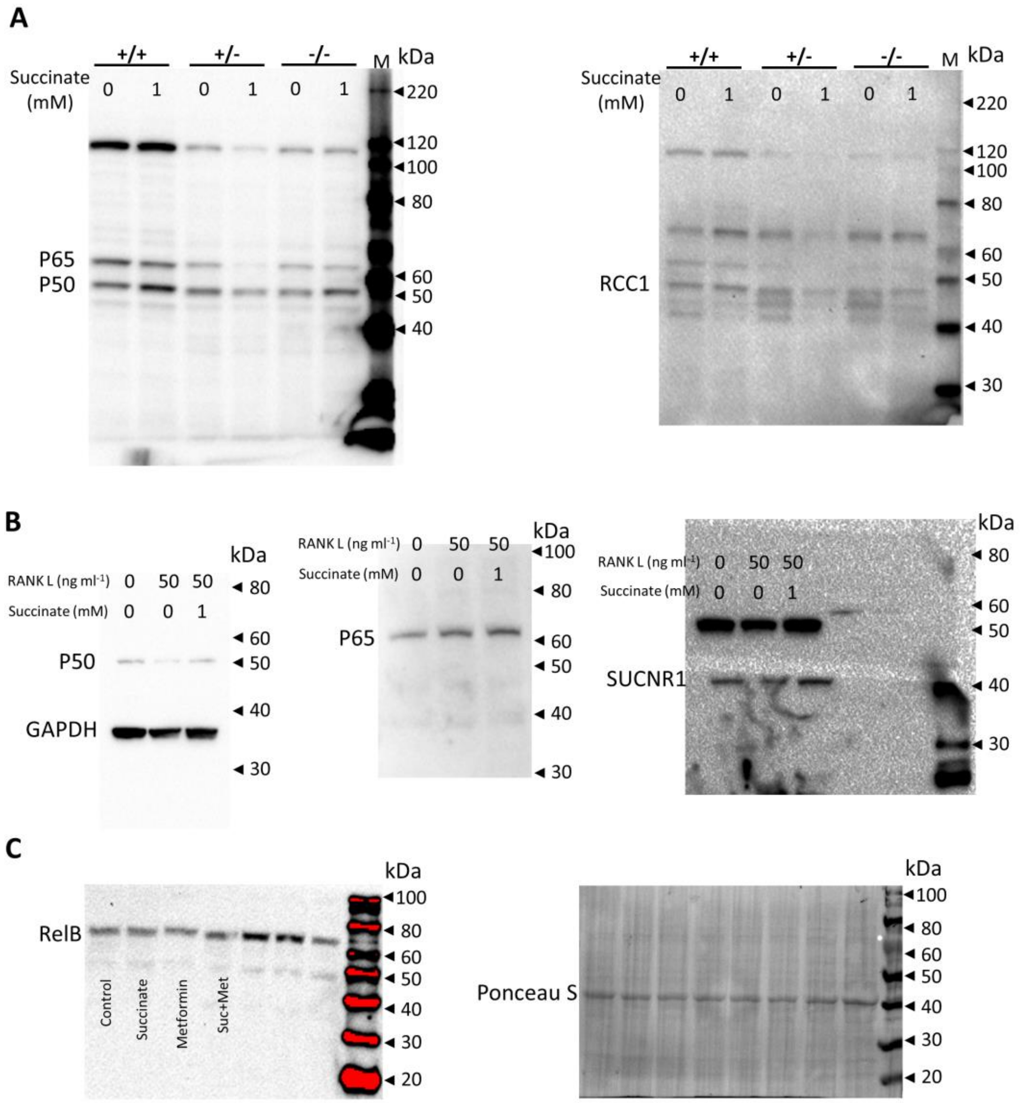

Supplementary Figure 9: Original western blot images used in Supplementary Figure 6. (**A**) Blots from Supplementary Figure 6A. (**B**) Blots from Supplementary Figure 6B. (**C**) Blots from Supplementary Figure 6C.

Supplementary Table 1. List of metabolites shown significant difference in MKR versus WT mice.

Metabolite	**MetID**	**Fold change***
Thiamine	C00378	175.27
Glucose 6-phosphate	C00092	51.60
Guanidineacetic acid	C00581	33.86
Succinic acid	C00042	**23.79**
Ornithine	C00077	18.73
N1-Acetylspermidine	C00612	18.25
Ergothioneine	C05570	17.78
Sedoheptulose 7-phosphate	C05382	16.76
1-Linoleoylglycerophosphocholine	C04100	13.15
Inosine	C00294	12.98
Histidine	C00135	12.86
Hypoxanthine	C00262	12.42
Tryptophan	C00078	12.35
Xanthine	C00385	11.79
Uridine	C00299	11.29
Methionine	C00073	11.21
N-Carbamoylputrescine	C00436	10.65
Cysteine	C00491	10.19
Tyrosine	C00082	10.07
Cinnamic acid	C10438	10.04
Isoleucine	C00407	9.07
Ribose 5-phosphate	C00117	9.01
Guanosine	C00387	8.98
Threonine	C00188	8.50
Linoleyl carnitine	HMDB06469	8.39
Phenylalanine	C00079	8.26
CMP	C00055	7.91
N-Acryloylglycine	HMDB01843	7.78
Sucrose	C00089	7.75
Carnitine	C00318	7.47
Putrescine	C00134	7.46
Carnosine	C00386	7.34
Serine	C00065	7.28
Gulonic acid	C00800	7.07
Proline	C00148	6.83
Adenosine	C00212	6.74
Cytidine	C00475	6.45
Lysine	C00047	6.31
Asparagine	C00152	6.21
Alanine	C00041	6.08
Cysteineglutathione disulfide	HMDB00656	6.02
Phosphocholine	C00588	5.99
UMP	C00105	5.72

Cytosine	C00380	5.63
GMP	C00144	5.62
Glutamine	C00064	5.60
Acetyl-L-serine	C00979	5.18
Creatine	C00300	5.07
Acetylcholine	C01996	4.89
Glycine	C00037	4.80
Glycerophosphocholine	C00670	4.74
Elaidic carnitine	HMDB06464	4.73
IMP	C00130	4.70
Homoserine	C00263	4.63
ADMA	C03626	4.62
2-(α-D-Mannosyl)-3-phosphoglycerate	C11516	4.53
Taurocyamine	C01959	4.52
Glyceraldehyde 3-phosphate	C00118	4.52
Aspartic acid b-semialdehyde	C00441	4.51
PC(16:0/0:0)	C04102	4.48
Palmitoyl-L-carnitine	C02990	4.35
PC(18:1(9Z)/0:0)[U] / PC(18:1(9Z)/0:0)[rac]	LMGP01050033	4.32
Arachidonic Acid	C00219	4.10
Stearoylcarnitine	HMDB00848	4.06
LysoPE	HMDB11489	4.01
N-Acetylserotonin	C00978	3.99
Acetylcarnitine	C02571	3.98
CDPcholine	C00307	3.92
Niacinamide	C00153	3.81
Pantothenic Acid	C00864	3.70
PC(20:4(5Z,8Z,11Z,14Z)/0:0)	LMGP01050048	3.69
Hypotaurine	C00519	3.68
Cyclic CMP	C00941	3.66
N2-Acetyl-L-ornithine	C00437	3.47
Methylcytidine	HMDB00982	3.46
Creatinine	C00791	3.36
Adenine	C00147	3.19
Choline	C00114	3.16
Valine	C00183	2.96
CDP-ethanolamine	C00570	2.82
DL-2-Aminoadipic acid	HMDB00510	2.73
Glutamic acid g-semialdehyde	C01165	2.70
Glyceric acid	C00258	2.68
Pyrrolidone-5-carboxylic acid	C02237	2.61
Propionyl-L-carnitine	C03017	2.57
N-Acetyl-L-alanine	HMDB00766	2.57
Malic acid	C00149	2.55
ADP-ribose	C00301	2.51
Glutamate	C00025	2.46

UDP	C00015	2.42
Hydroxylysine	C01211	2.37
Succinic acid semialdehyde	C00232	2.36
N-Acetylneuraminic Acid	C00270	2.35
Aminolevulinic Acid	C00430	2.32
CMP-N-acetylneuraminic acid	C00128	2.28
Aminoethylphosphonic acid	C03557	2.24
Pyrrole-2-carboxylic acid	C05942	2.23
Taurine	C00245	2.14
Glutathione, oxidized	C00127	2.10
Glucose	C00221	2.10
Trigonellinamide	C02918	2.07
Mannitol	C00392	2.06
AMP	C00020	1.98
UDP-D-galactose	C00052	1.94
Fumarate	C00122	1.93
Pyrroline hydroxycarboxylic acid	C04281	1.87
Uric acid	C00366	1.86
Hydroxy-L-glutamic acid	C03079	1.84
Cadaverine	C01672	1.80
UDP-N-acetyl-D-galactosamine	C00203	1.79
ADP	C00008	1.78
Phosphorylethanolamine	C00346	1.77
PS(22:1(11Z)/0:0)	LMGP03050023	1.75
Citrulline	C00327	1.71
Lactic acid	C01432	1.71
N-Acetyl-DL-methionine	HMDB11745	1.71
Hydroxyhydroquinone	C02814	1.70
Ne-Methyl-L-lysine	C02728	1.69
Phosphatidyl glycerol	C03274	1.68
Threonate	C01620	1.65
Aspartate	C00049	1.64
H2O4S	C00059	1.63
Oxaloacetate	C00036	1.62
Phosphoric acid	C00009	1.58
Selenium Sulfide	HMDB15106	1.57
D-Erythrose 4-phosphate	C00279	1.56
NAD	C00003	-1.55
Octanoylcarnitine	C02838	-1.55
D-Glycerate 3-phosphate	C00197	-1.82
PS(19:0/0:0)	LMGP03050028	-1.89
N-Acetylcadaverine	HMDB02284	-1.96
Oxoglutaric acid	C00026	-2.36
Glycerol	C00116	-2.73
Thymidine	C00214	-2.87
Methylhistamine	C05127	-3.16

Gamma-Butyrolactone	C01770	-3.26
Biotin	C00120	-3.58
L-Homocysteic acid	C16511	-6.17
Formylmethionine	C03145	-11.20
Guanine	C00242	-21.36
Serotonin	C00780	-24.00
Ascorbic acid	C00072	-37.72

N=4, * with Fold Change cutoff at 1.5-fold and p-value <0.01.)

Supplementary Table 2. List of metabolites significant changed by in metformin versus PBS treated MKR mice.

Metabolite	MetID	Fold change*
Guanine	C00242	19.74
Trigonellinamide	C02918	4.93
PC(24:0/0:0)	LMGP01050057	4.81
Thymidine	C00214	3.05
Uric acid	C00366	2.85
5-Aminoimidazole-4-carboxamide	C04051	2.52
Gulonic acid	C00800	2.41
Cer(d18:0/17:0)	LMSP02020022	2.08
N-Acetylcadaverine	HMDB02284	1.94
Cer(d18:0/16:0)	LMSP02020001	1.93
Threonate	C01620	1.58
CDPcholine	C00307	1.55
ADP	C00008	1.54
Glycerylphosphorylethanolamine	HMDB00114	1.54
Glycerol 2-phosphate	C02979	-1.54
Adenine	C00147	-1.54
AMP	C00020	-1.54
Niacinamide	C00153	-1.55
Glucose	C00221	-1.58
Inosine	C00294	-1.59
Succinic acid semialdehyde	C00232	-1.64
Acetylcholine	C01996	-1.70
Oxaloacetate	C00036	-1.71
Dihydrouracil	C00429	-1.71
ADP-ribose	C00301	-1.74
Propionyl-L-carnitine	C03017	-1.74
PC(18:1(9Z)/0:0)[U] / PC(18:1(9Z)/0:0)[rac]	LMGP01050033	-1.75
N-Acetyl-DL-methionine	HMDB11745	-1.77
Sarcosine	C00213	-1.78
Glyceraldehyde 3-phosphate	C00118	-1.80
Ne-Methyl-L-lysine	C02728	-1.82
Hydroxy-L-glutamic acid	C03079	-1.83
Aspartate	C00049	-1.93
Glycine	C00037	-2.01
Glutamic acid g-semialdehyde	C01165	-2.06
Adenosine	C00212	-2.11
N1-Acetylspermidine	C00612	-2.14
N-Acetyl-L-alanine	HMDB00766	-2.17
N2-Acetyl-L-ornithine	C00437	-2.20
Guanosine	C00387	-2.20
Proline	C00148	-2.36
N-Acetyl-L-glutamic acid	C00624	-2.39

Cysteineglutathione disulfide	HMDB00656	-2.39
Asparagine	C00152	-2.51
Homoserine	C00263	-2.52
Alanine	C00041	-2.53
Thiamine	C00378	-2.54
Ribose 5-phosphate	C00117	-2.62
Cyclic CMP	C00941	-2.63
Acetyl-L-serine	C00979	-2.70
Aspartic acid b-semialdehyde	C00441	-2.75
Palmitoyl-L-carnitine	C02990	-2.81
Lysine	C00047	-2.84
Tyrosine	C00082	-2.89
Ergothioneine	C05570	-3.03
Cysteine	C00491	-3.06
Linoleyl carnitine	HMDB06469	-3.11
Elaidic carnitine	HMDB06464	-3.14
Valineeg	C00183	-3.14
Tryptophan	C00078	-3.16
Phenylalanine	C00079	-3.21
Threonine	C00188	-3.42
Histidine	C00135	-3.43
Cinnamic acid	C10438	-3.44
Cytidine	C00475	-3.47
Serine	C00065	-3.50
Carnosine	C00386	-3.73
Methylhistamine	C05127	-3.86
Ornithine	C00077	-3.94
Glutamine	C00064	-4.14
Isoleucine	C00407	-4.30
ADMA	C03626	-4.70
N-Carbamoylputrescine	C00436	-4.74
Methionine	C00073	-5.51
Guanidineacetic acid	C00581	-5.70
N-Acryloylglycine	HMDB01843	-6.00
Sedoheptulose 7-phosphate	C05382	-6.33
Glucose 6-phosphate	C00092	-6.34
Succinic acid	C00042	-6.75
CMP	C00055	-6.94
Cytosine	C00380	-7.25

N=4, * with Fold Change cutoff at 1.5-fold and p-value <0.01.)

Supplementary Table 3. List of antibodies used in this study.

Antigen	Clone name	Host	Dilution	MW (kDa)	Source	Cat #
**Western Blot***						
P50	D7H5M	Rabbit monoclonal	1:1000	50	Cell Signaling	12540
P65	D14E12	Rabbit monoclonal	1:1000	65	Cell Signaling	8242
RelB	C1E4	Rabbit monoclonal	1:1000	70	Cell Signaling	4922
SUCNR1	A135	Rabbit Polyclonal	1:1000	38	Novus	NBP1-00861
RCC1	N/A	Rabbit Polyclonal	1:1000	45	Cell Signaling	3589
Histone H3	N/A	Rabbit Polyclonal	1:1000	17	Cell Signaling	9715
β-actin	N/A	Rabbit Polyclonal	1:1000	45	Cell Signaling	4967
GAPDH	D16H11	Rabbit monoclonal	1:1000	37	Cell Signaling	5174
**Indirect Immunofluorescence****						
P50	D7H5M	Rabbit monoclonal	1:200		Cell Signaling	12540
P65	L8F6	Mouse monoclonal	1:400		Cell Signaling	6956
Anti-rabbit IgG (H+L), F(ab')2 Fragment (Alexa Fluor® 488 Conjugate)			1:1000		Cell Signaling	4412
Anti-mouse IgG (H+L), F(ab')2 Fragment (Alexa Fluor® 594 Conjugate)			1:500		Cell Signaling	8890
DAPI		Blue fluorescent DNA dye	1:20000		Cell Signaling	4083
**Flow Cytometry*****						
CD11b	M1/70	Rat monoclonal	1:200		BD Pharmingen	553312
Ly6C	AL-21	Rat monoclonal	1:200		BD Pharmingen	561085

*For all the western blots, antibodies are diluted in 5% w/v BSA, 1X TBS, 0.1% Tween-20. **All the immunofluorescence antibodies are diluted in 5% w/v BSA, 1X TBS, 0.1% Tween-20. DAPI was diluted in 1X PBS. *** All the flow cytometry antibodies are diluted in 1X PBS with 2% w/v BSA.
